# *Brassica oleracea* L. var. *botrytis* Leaf Extract Alleviates Gentamicin-Induced Hepatorenal Injury in Rats—Possible Modulation of IL-1β and NF-κB Activity Assisted with Computational Approach

**DOI:** 10.3390/life12091370

**Published:** 2022-09-02

**Authors:** Hany Ezzat Khalil, Miada F. Abdelwahab, Promise Madu Emeka, Lorina I. Badger-Emeka, Al-Shaimaa F. Ahmed, Aliaa F. Anter, Sara Mohamed Naguib Abdel Hafez, Khalid A. AlYahya, Hairul-Islam Mohamed Ibrahim, Krishnaraj Thirugnanasambantham, Katsuyoshi Matsunami, Alyaa Hatem Ibrahim Selim

**Affiliations:** 1 Department of Pharmaceutical Sciences, College of Clinical Pharmacy, King Faisal University, Al-Ahsa 31982, Saudi Arabia; 2 Department of Pharmacognosy, Faculty of Pharmacy, Minia University, Minia 61519, Egypt; 3 Department of Biomedical Sciences, College of Medicine, King Faisal University, Al-Ahsa 31982, Saudi Arabia; 4 Department of Pharmacology and Toxicology, Faculty of Pharmacy, Minia University, Minia 61519, Egypt; 5 Department of Histology and Cell Biology, Faculty of Medicine, Minia University, Minia 61519, Egypt; 6 Department of Surgery, College of Medicine, King Faisal University, Al-Ahsa 36363, Saudi Arabia; 7 Department of Biological Sciences, College of Science, King Faisal University, Al-Ahsa 31982, Saudi Arabia; 8 Pondicherry Centre for Biological Science and Educational Trust, Puducherry 605004, India; 9 Department of Biotechnology, Saveetha School of Engineering, Saveetha Institute of Medical and Technical Sciences, Chennai 602105, India; 10 Department of Pharmacognosy, Graduate School of Biomedical & Health Sciences, Hiroshima University, 1-2-3 Kasumi, Minami-ku, Hiroshima 734-8553, Japan; 11 Department of Pharmacognosy, Faculty of Pharmacy, Sohag University, Sohag 82524, Egypt

**Keywords:** *Brassica oleracea*, cauliflower, gentamicin, GSH, AST, creatinine, IL-1β, NF-κB

## Abstract

Background: Recently, crop byproducts are considered a hot topic and can be converted into beneficial products. Cauliflower is well-known for its protective effects against oxidative stress-induced damage. The current study aimed to investigate the chemical profile and the ameliorative effects of cauliflower leaf extract (CL) on gentamicin-induced renal and hepatic injuries in rats. Methods: Cauliflower leaf was extracted with methanol to give the total methanol extract (TME) followed by the determination of total phenolic contents (TPC). Rats were divided into five groups; Group I was assigned as the control group, while the other groups were injected with gentamicin for ten days. Group II was given distilled water. Rats in groups III and IV were treated with oral CL (200 mg/kg and 400 mg/kg, respectively). Group V received L-cysteine (as a positive control). The functions of the kidneys and liver; oxidative stress and morphological and apoptotic changes of renal and hepatic tissues were assessed. Results: The TME was subjected to chromatographic techniques to yield ferulic acid, vanillic acid, p-coumaric acid and quercetin. TPC was 72.31 mg GAE/g of dried extract. CL treatment dose-dependently ameliorated gentamicin-induced impaired kidney and liver functions and improved the histopathological appearance of both organs. It also reduced gentamicin-induced oxidative stress. CL demonstrated downregulation of mRNA and protein expressions of IL-1β and NF-κB compared to nontreated rats. In silico interaction of the isolated compounds with amino acid residues of IL-1β and NF-κB might explain the current findings. Conclusion: Taken together, this study raises the waste-to-wealth potential of cauliflower to mitigate gentamicin-induced hepatorenal injury and convert the waste agromaterials into valuable products.

## 1. Introduction

Gentamicin (GTN) is a member of the aminoglycoside antibiotics, commonly indicated for the treatment of severe Gram-negative bacterial infections; however, nephrotoxicity and hepatotoxicity are reported as the major complications, which are primarily caused by reactive oxygen species [1,2,3,4]. Up to 50% of patients develop nephrotoxicity in therapeutic doses, while the toxic doses can cause permanent renal damage [5]. Kidneys are the organs that carry out a range of vital roles, including the removal and regulation of waste products and toxins from the blood [6]. The liver performs diverse essential functions involving metabolism and biotransformation, in addition to elimination and detoxification of xenobiotics or harmful substances [7,8]. Consequently, renal failure and liver toxicity will increase the risk for cardiovascular morbidity and mortality [6,9,10]. Despite the advances in the production of synthetic pharmaceuticals, the therapies established for the treatment of liver or kidney diseases remain sometimes of limited efficacy, show adverse effects and are often of high cost [7,9]. In the present days, the use of herbal medicines and the consumption of functional food or plant-derived bioactive metabolites as supplements has become more prominent to boost health and fight diseases [11]. In this context, the cruciferous vegetables including cauliflower, broccoli, Brussels sprouts and cabbage constitute dietary sources of phytoconstituents with potential health benefits [12,13]. They all belong to the family Brassicaceae (Cruciferae) or mustard family, which is broadly distributed, mostly in temperate regions, and includes around 338 genera and 3709 species. The family comprises economically interesting edible oil crops, e.g., canola or oilseed rape; condiment crops, e.g., mustard; and vegetable crops, e.g., cole crops (*Brassica oleracea*) [14,15]. Cauliflower (*Brassica oleracea* L. var. *botrytis*), rich in phytochemicals including glucosinolate and phenolic compounds such as hydroxylcinnamic acids such as ferulic, sinapic, caffeic and p-coumaric acids and their derivatives. It includes flavonoids and their derivatives. The isolated flavonoids are endowed with a large range of beneficial biological activities [16,17,18,19,20]. However, the leaf of cauliflower is not consumed and regarded as a postharvest waste product; evidence has shown that the leaf of cauliflower does contain some bioactive components as well and could be used as a source of antioxidants and could reduce food wastage [18]. Recently, the reutilization of the byproducts of edible vegetables has gained great scientific consideration, from the foregoing therefore, and based on the suggestion that the diversity of plant materials demonstrated the potential of hepatoprotective and nephroprotective activities through the reduction of oxidative stress [8,21,22,23,24]. The study was conducted to examine the activity of cauliflower leaf in GTN-induced kidney and liver injury in rats. In this regard, biochemical assays and histopathological observations of the liver and kidneys were carried out along with an assessment of liver and kidney functions and in silico studies. The study also enlightens the valorization of cauliflower leaf byproducts. In fact, this is very interesting from both industrial and medical perspectives.

## 2. Materials and Methods

### 2.1. General Procedures and Chemicals

Avance 400 NMR spectrometer was used to carry out the NMR analysis (^1^H-NMR was conducted at 400 MHz, while ^13^C-NMR was conducted at 100 MHz, Bruker, Switzerland). Silica gel 60 (Sigma-Aldrich, Darmstadt, Germany) was used to perform silica gel column chromatography (SCC). Diaion-HP-20 (Sigma-Aldrich, Darmstadt, Germany). Thin layer chromatography coasted with reversed phase silica gel (RPTLC) (Sigma-Aldrich, Darmstadt, Germany). Reversed phase column chromatography (RPCC) was carried out using C18 bounded silica gel of column chromatography type (Sigma-Aldrich, Darmstadt, Germany). Vanillin/sulfuric acid in ethanol (10%) and hotplate (150 °C) method was used for localization of spots. Freeze dryer (Millrock Technology, New York, NY, USA) was used to dry extract. High-performance liquid chromatography (HPLC) (Agilent, 1200 series, Waldbronn, Germany) using Discovery^®^ C18 column (5 cm × 4.6 mm × 5 μm) (Supelco, Bellefonte, PA, USA) was used. The mode of elution was isocratic using blend of methanol:acetonitrile:water (35:45:20, *v/v/v*) and injected at flow rate of 1.5 mL/min. The volume of injected sample was 10 μL (90 mg/mL, sample dissolved in methanol). Assay kits for malondialdehyde (MDA), glutathione (GSH) and superoxide dismutase (SOD) were purchased from Sigma-Aldrich, (St. Louis, MO, USA). Aspartate transaminase (AST), alanine transaminase (ALT) and alkaline phosphatase (ALP) kits were purchased from (Quimica Clinica Aplicada S.A, Amposta, Spain). All other chemicals were of analytical grade.

### 2.2. Plant Material

Cauliflower leaf (*Brassica oleracea* L. var. *botrytis*) was collected during October 2020, from a local market, Al-Ahasa, Saudi Arabia. Eng. Mamdouh Shokry, Zohria botanical garden, Giza, Egypt, kindly authenticated the plant material used in the current study. A voucher specimen (BOb-Oct-2020) was deposited in the herbarium of the Department of Pharmaceutical Sciences, College of Clinical Pharmacy, King Faisal University, Hofuf city, Kingdome of Saudi Arabia.

### 2.3. Extraction and Isolation of Plant Constituents

Cauliflower leaf (freeze-dried powdered plant material, 1.5 kg) was deeply extracted five times with 20 L of methanol (70%) for two weeks at room temperature. The compiled extract was concentrated using rotary evaporator to give the total methanol extract (TME) (dark green colored, 112.5 g). Fatty materials were removed from the TME (95 g) using petroleum ether, where the TME was suspended in one liter of distilled water and then partitioned with petroleum ether (7 times with 10 L) [25]. The compiled petroleum ether was concentrated to give petroleum ether fraction weighing 30 g. The remaining fraction (60 g) was subjected to CC applying Diaion HP-20 (1 kg) and eluted with water followed by different concentrations of methanol and water to produce the fractions of water (15 g), methanol (30%) (regarded as CLI, 5.3 g), methanol (50%) (regarded as CLII, 25.2 g) and methanol (100%) (regarded as CLIII, 14.1 g). Based on TLC pattern, the CLII fraction (25.2 g) was subjected to SCC (1000 g, using 8 L of CHCl_3_:CH_3_OH:H_2_O at ratio of (15:6:1) as mobile phase. The process resulted in seven main subfractions (CLII-1 to 7). Subfraction CLII-2 (2 g) was exposed to RPCC (250 g), implementing gradient elution using methanol:water as mobile phase), to give five main subtractions (CLII-2-1 to CLII-2-5). SubFr.CLII-2-2 (270 mg) was further purified by SCC and HPLC to yield vanillic acid (23 mg). SubFr.CLII-2-5 (60 mg) was purified using preparative RPTLC to yield p-coumaric acid (11 mg). Subfraction CLII-3 (4 g) was exposed to RPCC (400 g, implementing gradient elution using methanol:water as mobile phase) to give eight subtractions (CLII-3-1 to CLII-3-8). SubFr.CLII-3-6 (228.4 mg) was exposed to more purification applying preparative RPTLC and HPLC to yield the pure ferulic acid (14 mg) and pure quercetin (17 mg).

### 2.4. Calculation of Total Phenolic Content (TPC)

TPC was assessed applying the Folin–Ciocalteu index method detailed by Hany et al. [26] with modification. Stock solution of TME (1 mg/mL in methanol) was prepared. Half milliliter of Folin–Ciocalteu reagent and distilled water (6 mL) were successively mixed with stock solution (0.1 mL). Later, 20% sodium carbonate solution (1.5 mL) was additionally added to complete 10 mL volume. The media were kept for 2 h at room temperature. Then, for the different mixtures, absorbance was measured at 760 nm. Calibration curve of the standard (gallic acid) was prepared with the aid of serial dilutions of gallic acid.

### 2.5. Animals

Thirty Wistar rats (males, 200–220 g, aged 8–10 weeks) were obtained from the experimental animal facility at Nahda University in Beni Suef. Upon arrival, animals were kept under standard laboratory conditions and maintained on a 12 h light/dark cycle and at a controlled room temperature (27 °C) and were allowed acclimatization for two weeks. Animals were also allowed free access to food and water. The present study received the approval (Approval No. ES18/2020) of the Commission on the Ethics of Scientific Research, Faculty of Pharmacy, Minia University. Animals were assigned in random manner into 5 experimental groups of 6 animals per group after acclimatization.

### 2.6. Animal Experiment

Animals were assigned into 5 experimental treatment groups, and the study duration was 10 days. Group I represented the untreated control, which was given oral distilled water and i.p injection of saline daily. Group II was given GTN 100 mg/kg and oral distilled water. Group III was treated with CL 200 mg/kg + GTN, while Group IV was treated with CL 400 mg/kg + GTN. Group V was treated with L-cysteine (LC) one mmole/kg + GTN. Administration of GTN was by i.p route, whereas CL and LC were done orally [4]. At the end of the study, rats were anesthetized by sodium pentobarbital; then, blood was collected by cardiac puncture. Euthanasia was then performed by decapitation; thereafter, the kidneys and liver were harvested by carefully dissection. Harvested samples were then washed with phosphate-buffered saline (PBS) to get rid of any blood remaining in the tissues before being either stored at −80 °C for biochemical assessment or preserved in 10% formalin solution for processing of histological sections. The harvested tissues were used for biochemical estimation of antioxidant markers such as MDA, SOD and GSH. In addition, expressions of mRNA of interleukin-1β (IL-1β) and nuclear factor kappa B (NF-κB) were assayed.

### 2.7. Biochemical Analysis of Antioxidant Markers

Kidney and liver samples that were harvested in PBS (in a ratio of 1:10, *w/v*) were homogenized and followed by refrigerated centrifugation at 13,000× *g* for 30 min. Levels and contents of MDA, SOD and GSH were determined using the supernatant collected after centrifugation. MDA levels, a marker for lipid peroxidation, were determined according to the thiobarbituric acid reactive species (TBARS) method described earlier by Emeka et al. [27]. The levels of SOD and GSH were determined using the methods described and detailed by Khalil et al. [4].

### 2.8. Assessment of Kidney and Liver Functions

Collected blood samples were centrifuged at 2000 rpm for 15 min (with the use of re-frigerated centrifuge) to obtain serum samples. Thereafter, liver enzymes (ALT, AST and ALP), urea and creatinine levels were measured colorimetrically utilizing the methods used by routine Roche modular autoanalyzer equipment (Roche modular autoanalyzer, Tokyo, Japan) [28].

### 2.9. Kidney and Liver Histopathological Examinations

Fixed kidney and liver tissues were dehydrated in ascending concentrations of alcohol, cleaned with xylene and rapidly embedded in paraffin wax, which was sectioned at thickness of 5 µm. Subsequently, these sections were stained with H&E [29]. Olympus cx23ledrfs1 (Tokyo, Japan) light microscope was used to evaluate any apparent histopathological characteristics of prepared slides.

### 2.10. Morphometric Analysis of Renal and Hepatic Tissues

Evaluation of morphometric analysis was carried out at the power ×400 using Leica QWin 500 image analysis software (Leica Microsystems, Wetzlar, Germany). From each slide, ten nonoverlapping sections were used to calculate mean numbers of apoptotic cells and inflammatory cells (MNIC) [30].

### 2.11. Methods Used to Extract and Quantify mRNA

Fresh sections of harvested kidney and liver tissues were rinsed with ice-cold PBS; thereafter, they were used to extract the total mRNA using Trizol isolation reagent (Thermo Scientific, MA, USA). The extracted mRNA was quantified, and 300 ng/weight was used for cDNA determination for both apoptotic and inflammatory marker expressions. The forward primers (GAAATTCCTGATCCAGACAAAAAC, TGGACCTTCCAGGATGAGGACA and GCAAGGATACTGAGAGCAAGAG) and reverse primers (ATCACTTCAATGGCCTCTGTGTAG, GTTCATCTCGGAGCCTGTAGTG and GGATGGAATTGTGAGGGAGATG) for NF-κB, IL-1β and glyceraldehyde 3-phosphate dehydrogenase (GAPDH), respectively, were used. The expression of cDNA products was investigated using method detailed by Ibrahim et al. [31].

### 2.12. Western Blot Analysis

Treated tissues were used to extract the total protein lysate and quantified using Bradford quantification method. Western blot analysis was performed as described previously by Khalil et al. [32]. Primary antibodies against IL-1β (rabbit polyclonal antibody 1:750) (Biorbyt, Cambridge, UK), NF-κB (mouse monoclonal antibody 1:1200) (Invitrogen, Waltham, MA, USA) and β-actin (rabbit polyclonal antibody 1:1500) (Cell Signaling Technology, Beverly, MA, USA). HRP-labeled secondary antibodies (Biorybt, Cambridge, UK) and protein band signals were recorded using chemiluminescent reagents (Amersham Pharmacia Biotech, London, UK) and scanned using a densitometry scanner (Li-Cor, imaging system).

### 2.13. Computational Analysis

The docking assessment was performed applying the programs of Autodock tools (v1.5.4) and Autodock (v4.2). The structure of ligands was downloaded from PubChem database. Computational studies were carried out to explain the molecular basis underlying the interactions of VA (CID-8468), PCA (CID-637542), FA (CID-445858) and QRN (CID-5280343) ligands. The crystal structures of the IL-1β (PDB ID: 4G6M) and NF-κB (PDB ID: 3jv6) were downloaded from the Protein Data Bank (https://www.rcsb.org/) (accessed on 11 April 2022). The protein structure was prepared and optimized by the protein preparation modules in the assorted tools of the What IF server, PyMol and AutoDock software package, as described earlier by Khalil et al. [33]. The water molecules and crystal-bound molecules in the receptors were removed. The protein structures were protonated and optimized in physiological pH conditions by adding polar molecules. The AutoDock grid was centered around the cocrystallized ligand in a box size of 15 Å. Then, the docking module was used for docking the compounds into the IL-1β and NF-κB. Docked ligand–receptor interactions were visualized and analyzed using Discovery Studio 2021 Client trial version.

### 2.14. Statistical Analysis

Results obtained were expressed as mean ± S.E and analyzed with Graph Pad Prism (software V 8.2, San Diego, CA, USA). One-way analysis of variance was used to assess the comparisons between the control and treatment groups, while Tukey’s multiple comparisons test was used to test differences among the groups. At *p* values < 0.05, statistical difference was considered.

## 3. Results

### 3.1. Isolation and Identification of Secondary Metabolites

**Figure 1:**
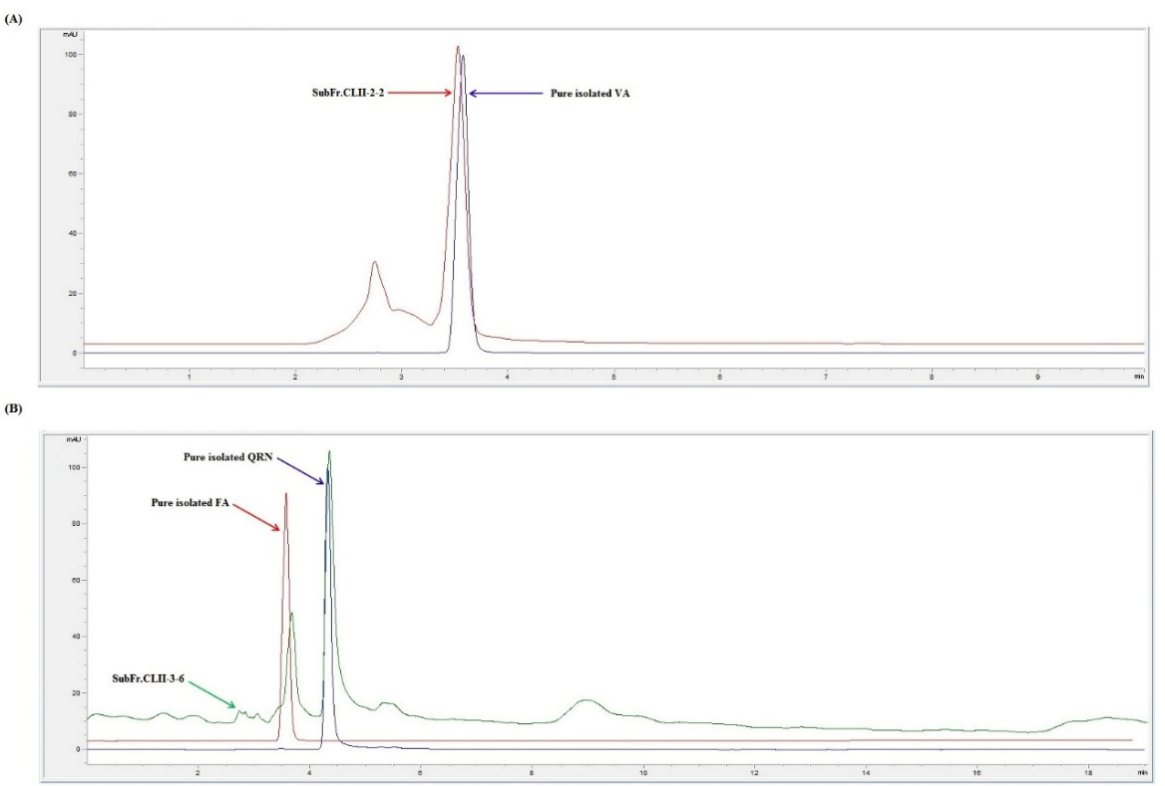
HPLC chromatograms of SubFr.CLII-2-2 (**A**) and SubFr.CLII-3-6 (**B**). Vanillic acid (VA), ferulic acid (FA) and quercetin (QRN).

**Figure 2:**
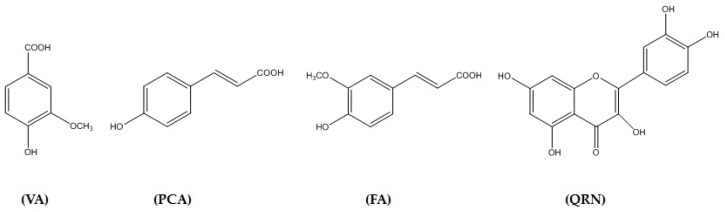
Structure of pure isolated constituents from cauliflower leaf. Vanillic acid (VA), p-coumaric acid (PCA), ferulic acid (FA) and quercetin (QRN).

The TME (50 g) was exposed to different chromatographic methods (Figure 1) to yield four pure compounds: vanillic acid (VA) [34], p-coumaric acid (PCA) [35], ferulic acid (FA) [35] and quercetin (QRN) [34]. The structures were explained by the examination of 1D- and 2D-NMR spectroscopic data including ^1^H, ^13^C, Distortionless Enhancement by Polarization Transfer (DEPT), Heteronuclear Multiple Quantum Coherence (HMQC) and Heteronuclear Multiple Bond Correlation (HMBC) (Figure 2, Supplementary Materials). The results were compared with those reported in the literature.

Vanillic acid: ^1^H-NMR (400 MHz, CD_3_OD): δ 7.58 (2H, br.s, H-2, 6), 6.83 (1H, d, *J* = 8.6 Hz, H-5) and 3.91 (3H, s, OCH_3_); ^13^C-NMR (100 MHz, CD_3_OD): δ 170.07 (COOH), 152.68 (C-3), 148.68 (C-4), 125.30 (C-1), 123.11 (C-6), 115.86 (C-2), 113.86 (C-5) and 56.44 (OCH_3_).

p-Coumaric acid: ^1^H-NMR (400 MHz, CD_3_OD): δ 7.62 (1H, d, *J* = 15.88 Hz, H-7), 7.44 (2H, d, *J* = 7.6 Hz, H-2, 6), 6.82 (2H, d, *J* = 7.6 Hz, H-3, 5) and 6.29 (1H, d, *J* = 15.88 Hz, H-8); ^13^C-NMR (100 MHz, CD_3_OD): δ 171.14 (C-9), 161.12 (C-4), 146.75 (C-7), 131.13 (C-2, 6), 127.30 (C-1), 116.87 (C-3, 5) and 115.64 (C-8).

Ferulic acid: ^1^H-NMR (CD_3_OD, 400 MHz): δ 7.61 (1H, d, *J* = 15.84 Hz, H-7), 7.19 (1H, br.s, H-2), 7.07 (1H, d, *J* = 8.08 Hz, H-6), 6.80 (1H, d, *J* = 8.08 Hz, H-5), 6.33 (1H, d, *J* = 15.84 Hz, H-8) and 3.91 (3H, s, OCH_3_); ^13^C-NMR (CD_3_OD, 100 MHz): δ 150.51 (C-3), 149.39 (C-4), 146.92 (C-7), 127.84(C-1), 123.99 (C-6), 116.51 (C-5), 115.96 (C-8), 111.77 (C-2) and 56.49 (OCH_3_).

Quercetin: ^1^H-NMR (DMSO-*d*_6_, 400 MHz): δ 12.49 (1H, br s, 5-OH), 7.69 (1H, s, H-2′), 7.55 (1H, d, *J* = 8.44 Hz, H-6′), 6.89 (1H, d, *J* = 8.44 Hz, H-5′), 6.41 (1H, s, H-8) and 6.20 (1H, s, H-6); ^13^C-NMR (DMSO-*d*_6_, 100 MHz): δ 175.80 (C-4), 163.85 (C-7), 160.69 (C-5), 156.11 (C-9), 147.66 (C-4′), 146.77 (C-2), 145.01 (C-3′), 135.69 (C-3), 121.94 (C-1′), 119.95 (C-6′), 115.57 (C-5′), 115.05 (C-2′), 102.98(C-10), 98.15 (C-6) and 93.32 (C-8).

### 3.2. TPC

TPC was considered as the equivalence of milligrams of gallic acid per gram of dried plant (mg GAE/g). TPC was found to be 72.31 mg GAE/g of dry extract. The value was based on the regression equation (y = 0.169x − 0.0053, R^2^ = 0.9578).

### 3.3. Effects of CL on the Levels/Contents of MDA, GSH and SOD

**Figure 3:**
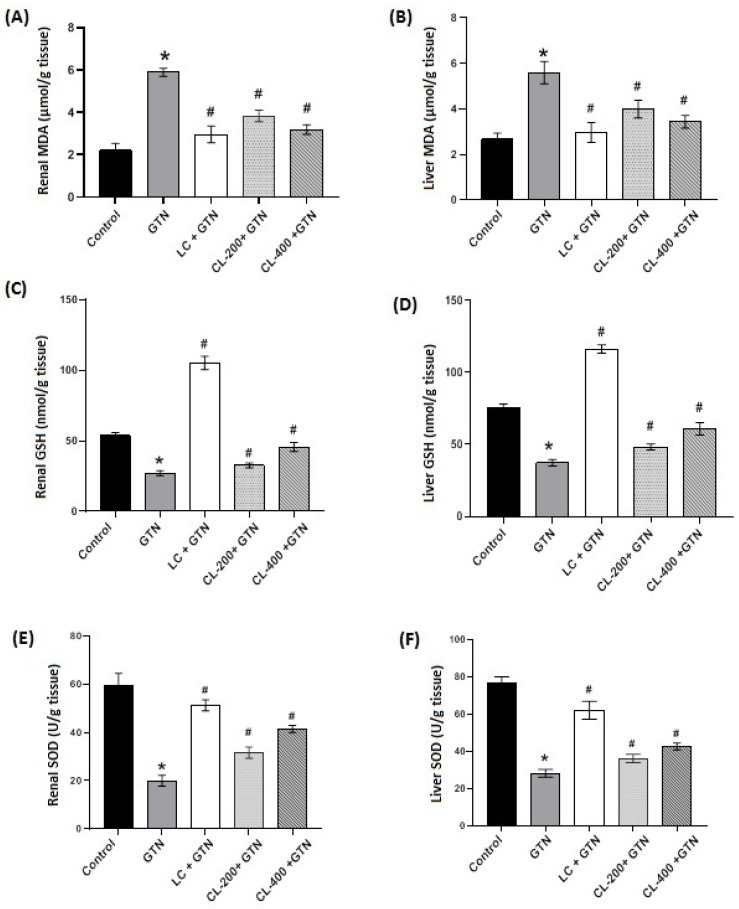
The effects of GTN alone or in combination with CL (200 or 400 mg/kg) or L-cysteine on renal and hepatic oxidative stress status. (**A**) Bar charts showing the effect of GTN and different treatments of renal tissue levels MDA. (**B**) Liver tissue levels of MDA. (**C**) Renal tissue GSH content, and (**D**) represents GSH content in the liver. SOD in renal tissue (**E**) and SOD in liver tissue (**F**). Data are presented as mean ± SE. * represents a significantly (*p* < 0.05) different comparison with the control; # indicates significant (*p* < 0.05) difference between treatment with GTN only and treatments with CL doses and LC.

The results show significantly increased (*p* < 0.05) levels in the renal lipid peroxidation measured as MDA with GTN treatments compared to the control. However, in the case of the doses of CL in combination with GTN and LC + GTN treatments, MDA levels were significantly (*p* < 0.01) decreased compared to the GTN group. In addition, there was no difference between CL (200 and 400 mg/kg) plus GTN and LC + GTN treatments compared to the control (Figure 3A). Similar trends were observed in the liver tissue MDA level as seen with the renal MDA level (Figure 3B). Treatment with CL 400 mg/kg + GTN and LC + GTN showed comparable levels. The GSH content of renal tissue as presented in Figure 3C shows that the GTN-treated group significantly reduced renal tissue GSH content. On the other hand, oral administration of CL (200 and 400 mg/kg) resulted in significantly (*p* < 0.05) increased GSH levels, which were decreased in comparison with the control group. The LC + GTN-treated group produced a much more marked reduction in GSH content since LC is a natural precursor of GSH. The GSH content of liver tissue showed a similar profile both with GTN induction and treatments with CL (200 and 400 mg/kg). Comparatively, treatment with LC also boosted GSH content much more compared to the control as shown in Figure 3D. Figure 3E,F represent renal and liver tissue SOD activities. Our findings indicate that there is a markedly (*p* < 0.05) reduced activity of renal tissue SOD induced by GTN treatment in relation to SOD activity of the control group. In all the other treatment groups, SOD activities increased in a significant fashion in renal and liver tissues compared to the groups treated with GTN. In both tissues, the LC + GTN treatment resulted in higher SOD activities. Although CL (200 and 400 mg/kg) + GTN treatments show a significant increase in SOD activity compared to GTN alone, they did not completely reverse the effect of GTN.

### 3.4. Effect of Caulifower Leaf Extract on Kidney Function Parameters

**Figure 4:**
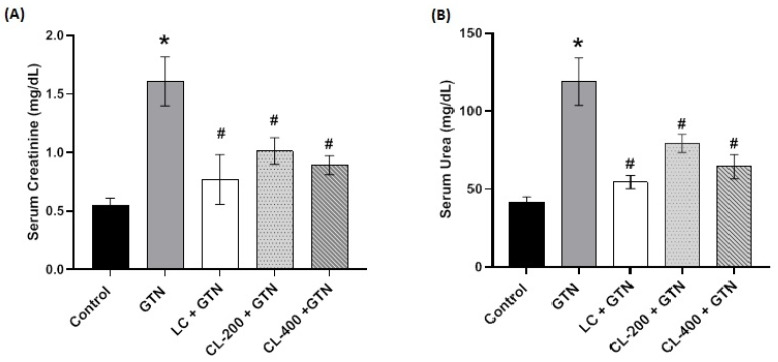
The effects of GTN alone or in combination with CL (200 or 400 mg/kg) or L-cysteine on kidney function parameters. (**A**) Bar charts showing the effect of GTN and different treatments on serum creatinine, while (**B**) shows that for serum urea. Data are presented as mean ± SE. * rep-resents a significantly (*p* < 0.05) different comparison with the control; # indicates a significant (*p* < 0.05) difference between GTN-treated group and other treatment groups.

Kidney function was assessed by the determination of serum creatinine and urea. Serum creatinine and urea in the different experimental groups are shown in Figure 4A,B. The results indicate that there was a significant increase in the serum creatinine level with GTN treatment. However, the treatment groups of 200 and 400 mg/kg of CL in combination with GTN were found to ameliorate the levels of serum creatinine bringing them to the level of the control. This reduction was significant compared to the GTN group. In addition, for the serum urea, the GTN + CL (200 and 400 mg/kg)-treated groups showed a significantly decreased level compared to the group treated with GTN only.

### 3.5. Effect of Caulifower Leaf Extract on Liver Function

**Figure 5:**
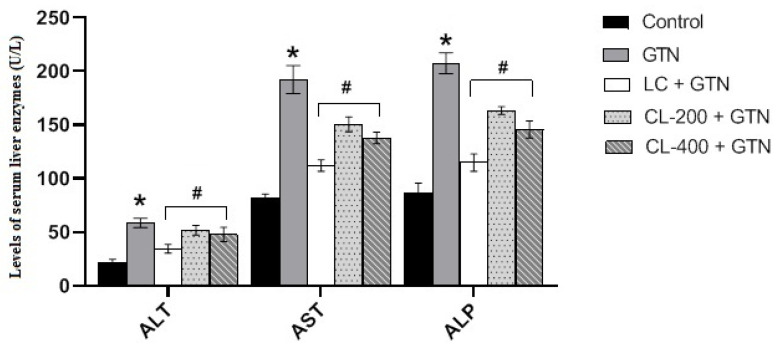
The effects of GTN alone or in combination with CL (200 or 400 mg/kg) or L-cysteine on liver function parameters. Bar charts showing the effect of GTN and different treatments on the levels of ALT, AST and ALP (liver enzymes). Results represent mean ± S.E. * represents a significantly (*p* < 0.05) different comparison with the control; # indicates a significant (*p* < 0.05) difference between treatments with GTN group, CL group and LC group.

The liver function was assessed by determination of serum liver enzymes. Figure 5 shows the liver enzyme levels of AST, ALT and ALP in rats treated with GTN alone or in combination with CL (200 and 400 mg/kg) or LC. Our results reveal that GTN treatment increased the liver enzyme levels of ALT, AST and ALP significantly (*p* < 0.05) compared to the control group. However, the administration of CL and LC to rats in the presence of GTN significantly prevented GTN elevation of liver enzymes.

### 3.6. Histopathological Analysis of Renal Tissue

**Figure 6:**
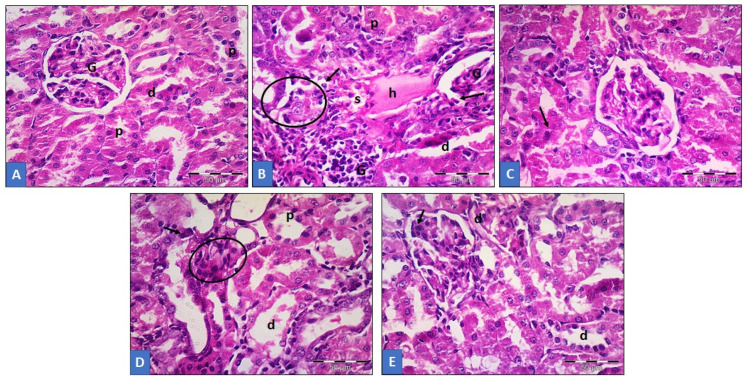
Showing histology of renal tissue presented as photomicrographs. (**A**) Control group displaying normal cellular morphological organization of the renal tissue with normal glomeruli capillary tufts (G), narrow-lumen PCTs (p), surrounded by acidophilic cytoplasm, with wide-lumen DCTs (d) with cytoplasm having less acidophilic outlook and an arrow showing visible vesicular nuclei. (**B**) Group treated with GTN displaying distorted lobular cell structure architecturally, with infiltration of inflammatory cells (circle) with atrophic glomeruli (G), PCTs (p) and DCTs (d) appeared dilated with lining the tubules showing numerous apoptotic cells as indicated by arrows. The presence of hyaline cast (h) and intratubular debris (s) were also observed. (**C**) is treatment with CL 200 mg/kg showing less distorted renal cellular architecture, which appeared very improved histologically, few apoptotic cells (arrow), less distorted lobular structure with tubules that are dilated (p,d) and cell infiltration of inflammatory cells represented as a circle. (**D**) represents treatment with a higher dose (400 mg/kg) of CL with appearance of near-normal cellular tissue. However, few tubules appear dilated (d), less infiltration of cells by inflammatory cells indicated as circle. (**E**) rat renal tissue treated with LC revealing that the nephrons appeared normal with restored cellular structures, although there are visible apoptotic cells, which are few (arrow).

Histological photomicrographs analysis of renal tissue after treatments with GTN, CL (200 and 400 mg/kg) and LC (Figure 6). Figure 6A represents rat renal tissues that served as the control treatment having no pathological morphology, hence showing normal cellular/lobular structure and an organized appearance. Overlaps of the glomeruli by capillary tufts were present. Narrowed lumen of the proximal convoluted tubules (PCTs) with high acidophilic cytoplasm were observed. In addition, it also showed wide-lumen distal convoluted tubules (DCTs), however, with vesicular nuclei and less acidophilic cytoplasm. Figure 6B represents treatment with GTN displaying distorted lobular architecture with cellular infiltration of the inflammatory cells. The glomeruli appeared atrophic having dilated PCTs and DCTs. Very visibly, many apoptotic cells were seen lining the tubules, and intratubular debris having a hyaline cast was also prominently observed. Figure 6C shows the CL 200 mg/kg-treated group with improved renal architecture but with some focal areas having dilated tubules. Figure 6 represents the CL 400 mg/kg-treated group with well-improved renal architecture, also with very few disturbed lobular organizational structures. The glomeruli were seen replaced by inflammatory cells. Notice the dilated tubules with numerous apoptotic cells. Figure 6E shows rats treated with LC, exhibiting the restoration of normal lobular organization of the renal tissue but with few apoptotic cells (Figure 6).

### 3.7. Histopathological Analysis of Liver Tissue

**Figure 7:**
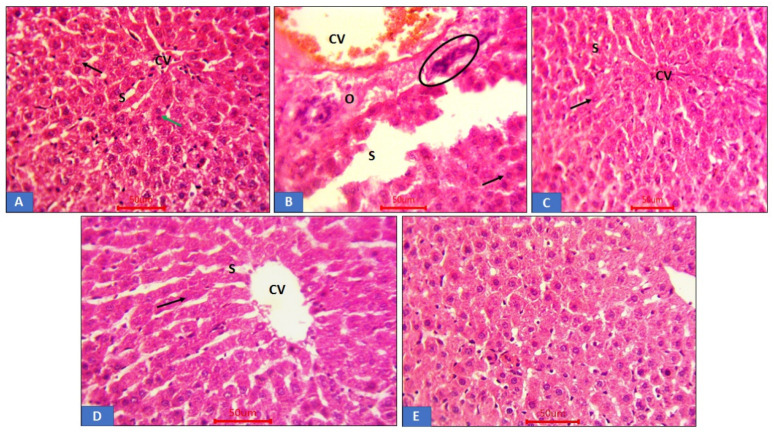
Displaying histopathology images of rat hepatic tissue. (**A**) Control hepatic tissue depicting normal hepatic architecture showing a central blood vein (CV) with cords of hepatocytes (arrow), granular cytoplasm and large rounded vesicular nuclei separated by the blood sinusoids (S). It also shows hepatocytes that are binucleated (green arrow). (**B**) GTN-treated group was seen with distorted hepatic cell architecture. In addition, it shows dilated blood central vein (CV) and sinusoids (S) with inflammatory cell infiltration (circle) and oedema (O). (**C**) (CL 200 mg/kg) shows minor blood sinusoid (S) dilation with apoptotic cells (arrow) that are few. (**D**) CL dose of 400 mg/kg-treated group showing near-normal hepatic cellular structure. (**E**) LC group showing more or less normal structure with central blood vein (CV) with sinusoids (S) and hepatic cells (arrow).

The results (Figure 7) show the histological photomicrograph of liver tissue treated with GTN and cauliflower extracts. Figure 7A shows the control-treated group having normal hepatic cellular architecture with central vein and granular cytoplasm. It also had rounded vesicular nuclei that appear large, which were separated by blood sinusoids with binucleated hepatic cells. Figure 7B is the group treated with GTN showing distorted hepatic cells. Dilated blood veins and sinusoids with oedema and inflammatory cells infiltration were observed. Figure 7C,D show CL-treated groups with normal hepatic tissue architecture; however, CL 200 mg/kg displayed both dilated central veins and blood sinusoids. A few hepatocytes lost their nuclei shown as darkly stained hepatic nucleoli with slight dilated central vein. Furthermore, the CL + GTN group exhibited a more normal structure, central vein, blood sinusoids and hepatocytes compared to control. Figure 7E represents LC treatments with near-normal hepatic architecture and normal granular cytoplasm.

### 3.8. Morphometric Analysis of Renal and Liver Tissues

**Figure 8:**
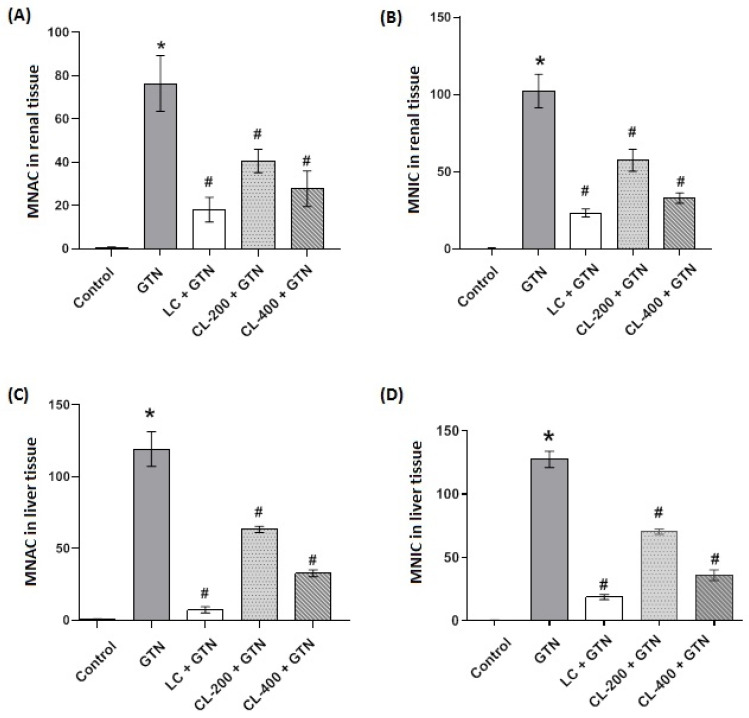
The effects of GTN alone or in combination with CL (200 or 400 mg/kg) or LC on the number of apoptotic and inflammatory cells in kidney and liver tissues. Bar charts showing morphometric result analysis of the effect of GTN and different treatments of renal and liver tissue sections. (**A**) shows renal tissue MNAC, while (**B**) represents renal tissue MNIC. (**C**) shows MNAC in liver tissue, while (**D**) represents liver tissue MNIC. Data are presented as mean ± SE (n = 6). Significantly variant (*p* < 0.05). * represents a significantly (*p* < 0.05) different comparison with the control; # indicates a significant (*p* < 0.05) difference between GTN-treated group and other treatment groups. MNAC = mean number of apoptotic cells; MNIC = mean number of inflammatory cells.

Morphometric results analysis of GTN-treated group, show significantly (*p* < 0.05) increased numbers of renal and liver apoptotic tissues as well as inflammatory cells. However, as indicated in Figure 8A–D, CL treatments (200 and 400 mg/kg) significantly (*p* < 0.05) reduced the effects of GTN by decreasing the apoptotic and inflammatory cell numbers in the kidneys and liver. However, LC treatment showed a better protection of both kidney and liver cells by significantly (*p* < 0.05) reducing apoptotic and inflammatory cell changes caused by GTN treatments.

### 3.9. Effect of Caulifower Leaf Extract on mRNA and Protein Expressions of IL-1β and NF-κB Markers in Renal and Liver Tissues

**Figure 9:**
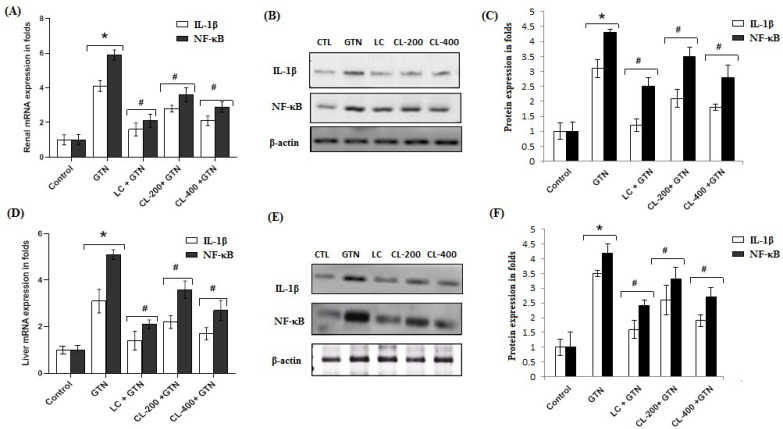
The effects of GTN alone or in combination with CL (200 or 400 mg/kg) or L-cysteine on renal and hepatic mRNA and protein expression of IL-1β and NF-κB. Treatments with GTN, CL doses of 200 and 400 mg/kg and LC on IL-1β and NF-κB markers were quantified using quantitative real-time PCR. GAPDH was used as an internal mRNA control, for renal tissue (**A**) and for liver tissue (**D**). Alterations in the status of protein expression in response to GTN, CL (200 and 400 mg/kg) and LC were inspected using Western blot. β-Actin was utilized as a control, for renal tissue (**B**,**C**) and for liver tissue (**E**,**F**). The experimental data are shown as the mean ± SE of triplicate values; * represents a significantly (*p* < 0.05) different comparison with the control; # indicates a significant (*p* < 0.05) difference between treatments with GTN group, CL group and LC group.

Figure 9 shows renal and hepatic determination of mRNA expressions of inflammatory markers (IL-1β and NF-κB). Renal mRNA and protein expressions of inflammatory markers, IL-1β and NF-κB, were markedly (*p* < 0.05) elevated in the GTN-treated group compared to the control (Figure 9A–C). Figure 9D–F show hepatic mRNA and protein expressions of IL-1β and NF-κB showing a similar pattern to that of renal expressions. The results show that NF-κB expression appeared to be more in GTN-treated groups as compared with the control group of renal and liver tissues, respectively. Treatments with CL 200 and 400 mg/kg doses in GTN pretreatment produced a significant (*p* < 0.05) decrease in both mRNA and protein expressions of IL-1β and NF-κB in a dose-dependent manner in both tissues. 

### 3.10. In Silico Binding of VA, PCA, FA and QRN with NF-κB and IL-1β Markers

**Figure 10:**
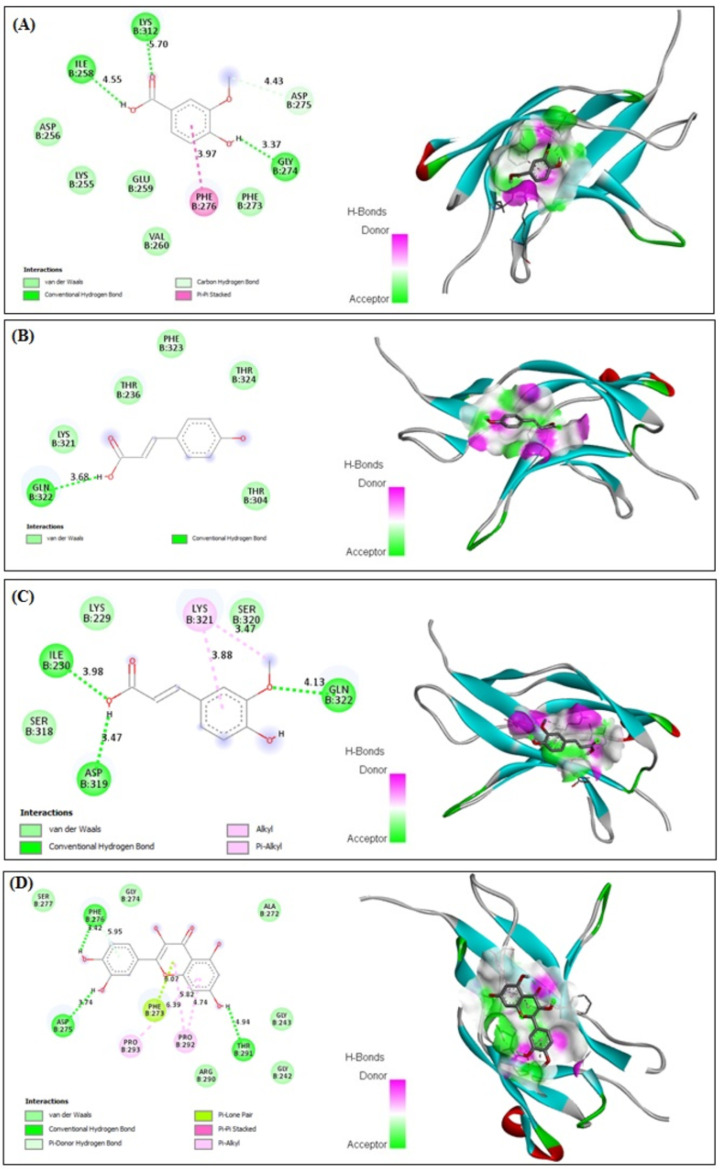
In silico analysis showing (docking, amino acid binding interactions and binding pocket of ligand–receptor interactions) of VA (**A**), PCA (**B**), FA (**C**) and QRN (**D**) with NF-κB marker. Vanillic acid (VA), p-coumaric acid (PCA), ferulic acid (FA), quercetin (QRN) and nuclear factor kappa B (NF-κB).

**Figure 11:**
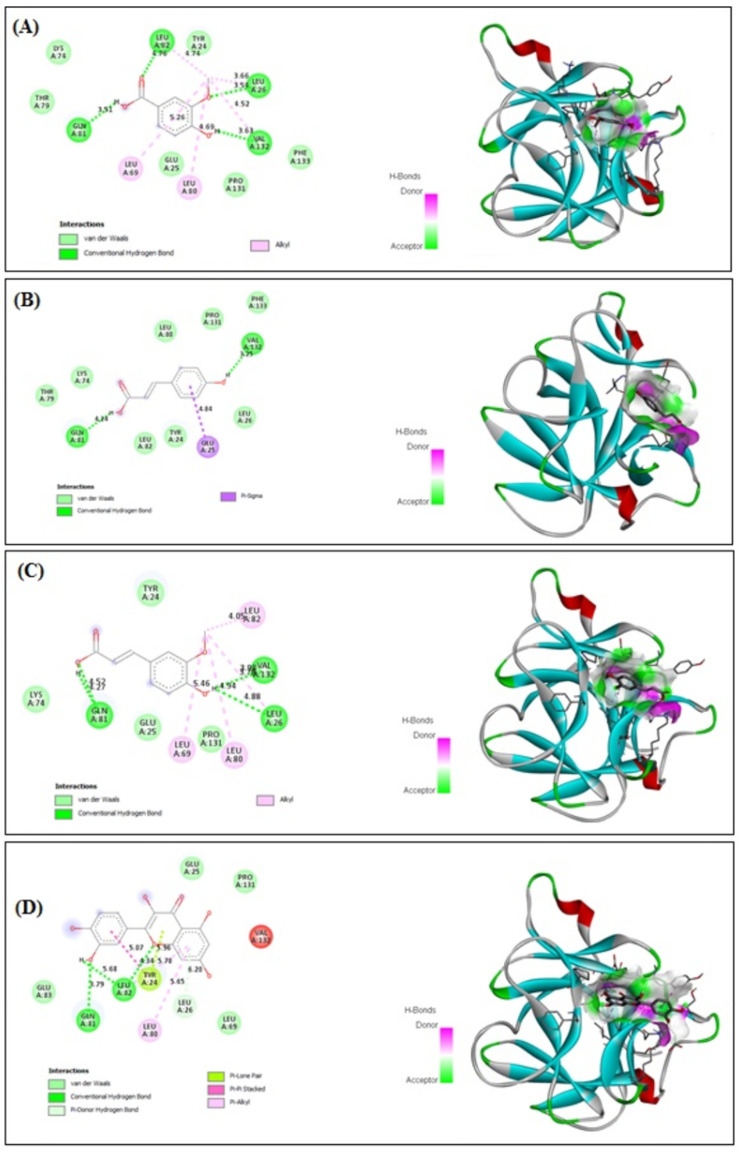
In silico analysis showing (docking, amino acid binding interactions and binding pocket of ligand–receptor interactions) of VA (**A**), PCA (**B**), FA (**C**) and QRN (**D**) with IL-1β marker. Vanillic acid (VA), p-coumaric acid (PCA), ferulic acid (FA), quercetin (QRN) and interleukin-1β (IL-1β).

VA, PCA, FA and QRN showed improved binding affinity with NF-κB (−4.27, −3.35, −3.84 and −5.63 kcal/mol, respectively) and strong binding affinity with IL-1β (−5.19, −5.33, −5.71 and −5.43 kcal/mol, respectively). The receptors showed stable binding with all ligands (VA, PCA, FA and QRN) through the formation of various interactions. Interestingly VA formed four kinds of interactions: van der Waals, conventional hydrogen, carbon hydrogen and Pi–Pi stacked interactions (Figure 10A) with NF-κB and van der Waals, conventional hydrogen and alkyl interactions with IL-1β (Figure 11A). On the other hand, PCA formed two types of interactions: van der Waals and conventional hydrogen interactions (Figure 10B) with NF-κB and van der Waals and conventional hydrogen and Pi–sigma interactions with IL-1β (Figure 11B). FA demonstrated four types including van der Waals, conventional hydrogen, Pi–alkyl and alkyl interactions (Figure 10C) with NF-κB and van der Waals, conventional hydrogen and alkyl interactions with IL-1β (Figure 11C). QRN formed six types of interactions comprising van der Waals, conventional hydrogen, Pi–lone pair, pi–pi stacked, Pi–donor hydrogen and Pi–alkyl interactions (Figure 10D) with NF-κB and the same kind of interactions with IL-1β (Figure 11D). Most of the reductions of energies occurred due to hydrogen interactions, thus improving the binding strength between the ligands and receptors. These findings reveal that all isolated compounds VA, PCA, FA and QRN are highly competitive agonists of NF-κB and IL-1β.

## 4. Discussion

GTN is an effective antimicrobial agent that has been used to treat serious infections caused by Gram-negative bacteria.

However, due to its serious side effects on kidney and liver functions, its clinical usefulness is therefore limited [4]. Oxidative stress is described as an imbalance between oxidant activity in terms of increased reactive oxygen species usually called free radicals and antioxidants acting as cell protectors in different tissues. Drug-induced oxidative stress will produce these free radicals faster than the protecting antioxidants resulting in oxidative stress. This phenomenon is due to the upregulation of proinflammatory signaling genes such as NF-kB and cytokines causing tissue damage [36,37]. Specifically, GTN-induced toxicity has been linked to the induction of oxidative stress and a consequent inflammation leading to cell apoptosis [35,38]. Therefore, the activities of CL (200 and 400 mg/kg) on the attenuation of GTN-induced oxidative stress, inflammation and apoptosis were evaluated in the present study. Cauliflower belongs to the cruciferous (Brassicaceae) family of vegetables, which has been reported to possess both antioxidant and inflammatory activities [16,39,40,41]. Studies have shown that its antioxidant activity is attributed to its flavonoids and phenolic acid contents along with antioxidant vitamins [17]. The present study revealed the isolation and identification of four compounds from the TME of the CL extract. They include three major pure phenolic acids and one flavonoid. From spectral data based on ^1^H, ^13^C, DEPT, HMQC and HMBC, the three phenolic acids were identified as vanillic acid, p-coumaric acid and ferulic acid, while the flavonoid was identified as quercetin (Figure 2 and Supplementary Materials).

The current finding of TPC aligns with those of reported values [24]. The higher TPC was also observed to have an abundance of the isolated pure phenolic compounds from the TME as previously mentioned. These phenolic acids have been previously shown to ameliorate GTN-induced oxidative stress [42,43,44]; thus, this study aimed to assess cauliflower leaf byproducts against GTN-induced toxicity. The present study findings show that GTN induced both kidney and liver dysfunction, increased tissue lipid peroxidation, measured as levels of lipid peroxidation, but treatment with CL (200 and 400 mg/kg) decreased this effect indicating an antioxidant effect of the extract. Our observation is similar to that reported by other studies [16,24], which documented that cauliflower extracts exhibited free radical scavenging activity. GSH and SOD activities were reserved in rats treated with CL (200 and 400 mg/kg) + GTN emphasizing its effect against oxidative stress. As reported previously, GTN leads to elevated serum creatinine and urea levels by causing renal parenchymal injury [45,46]. Consistent with these reports is the study performed by Huang et al. [47] on mice showing high levels of serum creatinine and urea induced by GTN. These results are in accordance with our present study. Treatments with CL (200 and 400 mg/kg) reduced serum creatinine and urea, and this effect was similar to the effect of LC, which is a known precursor of GSH, with the ability to neutralize reactive molecules that cause cell and tissue damage [48]. Accumulating evidence has shown that the administration of GTN to experimental animals produced a profound elevation in serum AST and ALT indicating liver injury [4,49,50]. These elevations in liver enzymes by GTN were reproduced in the present study; however, treatment with cauliflower extracts showed a reduction in ALT, AST and ALP. Noteworthy, treatment with LC exhibited a better effect on the reduction profiles of liver enzymes induced by GTN treatments. Therefore, a possible liver injury protection can be offered by these extracts. The presence of oxidative stress caused by reactive oxygen species also promotes inflammation. Histopathological examination of the kidney tissues revealed a distorted lobular cellular morphology showing infiltration of inflammatory cells with atrophic glomeruli having dilated PCT and DCT tubules. In addition, numerous apoptotic cells were observed lining the tubules. The liver sections showed distorted hepatic cellular structure with a dilated central blood vein, and sinusoids with edema and infiltration of inflammatory cells observed.

Our results reveal that treatment with cauliflower extracts showed amelioration of the renal and liver histological structures and reduced inflammatory cell infiltration. These results confirm the ability of CL extract to protect the kidneys and the liver against GTN-induced damage. It is believed that oxidative stress can trigger a variety of differential gene expressions, which are involved in inflammation. Drugs such as GTN reported to initiate the inflammatory process leading to increased expressions of proinflammatory cytokines [51]. The induction of IL-1β is reported to be regulated by the NF-κB-activated signaling pathway [52]. On the other hand, IL-1β has been documented to increase the activity of NF-κB once activated [53,54]. In the present study, we observed that CL (200 and 400 mg/kg) suppressed GTN-induced mRNA and protein expressions of proinflammatory markers (NF-κB and IL-1β) in both the kidneys and liver. Interestingly, our data are in agreement with a study by Larocca et al. [55], who showed that cauliflower leaf reduced a lipopolysaccharide-induced increase in proinflammatory cytokines in rabbits. Evidence that GTN administration causes necrosis, inflammation and apoptosis in the kidneys and liver is well-documented [56,57]. A computational analysis revealed that compounds present and isolated from TME (VA, PCA, FA and QRN) showed binding and interaction with the amino acid residues of NF-κB and IL-1β, which might provide an explanation of the observed protective effect of CL extract against renal and hepatic injuries induced by GTN. Taken together, the present results clearly indicate that CL possesses the potential to mitigate the adverse effects caused by the administration of GTN via its antioxidant and anti-inflammatory properties demonstrated in this study. Consequently, cauliflower leaf byproduct could potentially be utilized to produce new functional foods and medication.

## 5. Conclusions

The conducted study provided insights into the contribution of the chemical profile and bioactivity of the leaf agrofood byproducts of cauliflower. The study demonstrated the isolation and identification of vanillic acid, p-coumaric acid, ferulic acid and quercetin. The results show that CL treatments mitigated GTN-induced lipid peroxidation, via the reduction of MDA, in addition to improving renal and liver SOD levels as well as GSH contents. Furthermore, liver and kidney function parameters were restored to normal levels by CL treatments. Histopathological assessment also revealed restored features of kidney and liver tissues by the administration of CL. GTN-induced elevation of IL-1β and NF-κB markers was downregulated by CL treatment. The potent binding of IL-1β and NF-κB amino acid residue with vanillic acid, p-coumaric acid, ferulic acid and quercetin further supports the results of this study. The study suggests a future deployment of an ecofriendly strategy for the investment of agroindustrial leaf waste of cauliflower to develop functional food candidates with high antioxidant capacity.

## Data Availability

The data presented in this study are available on request from the corresponding author.

## Supplementary Materials

The following supporting information can be downloaded at: https://www.mdpi.com/article/10.3390/life12091370/s1: Supplementary. 1D and 2D-NMR spectroscopic data of vanillic acid, p-coumaric acid, ferulic acid and quercetin.
