# Supplementary material for: Brassica oleracea L. var. botrytis Leaf Extract Alleviates Gentamicin-Induced Hepatorenal Injury in Rats—Possible Modulation of IL-1β and NF-κB Activity Assisted with Computational Approach
Source: Life (Basel). 2022 Sep 2;12(9):1370. doi: 10.3390/life12091370 (PMC9504091; doi:10.3390/life12091370)
Supplement: Supplementary file 1 [file life-12-01370-s001.zip › Supplementary‎-S.pdf]

Supplementary-S: 1D- and 2D- NMR spectroscopic data of pure isolated compound

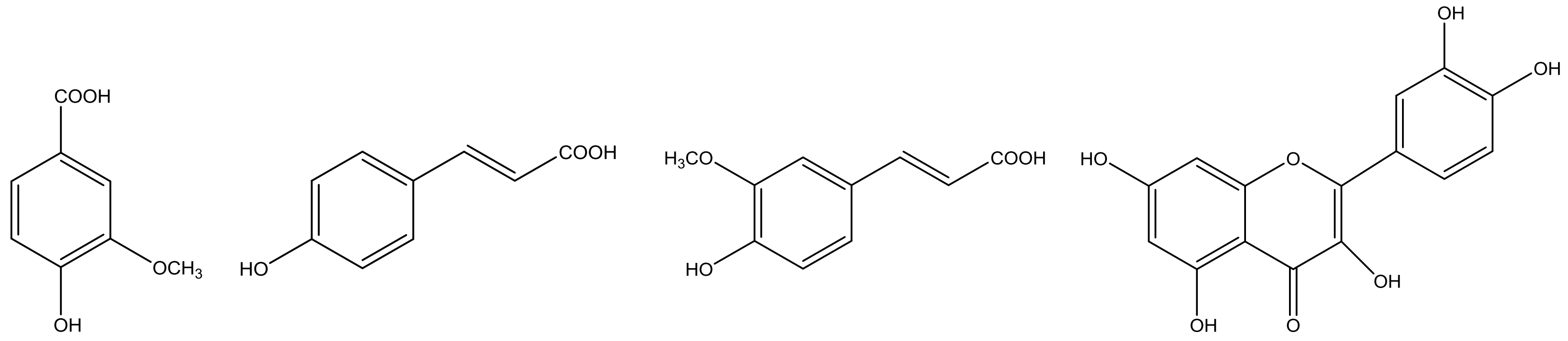

(VA) (PCA) (FA) (QRN)

Structures of pure isolated constituents from cauliflower leaf .
Vanillic acid (**VA**), p-coumaric acid (**PCA**), ferulic acid (**FA**) and quercetin (**QRN**).

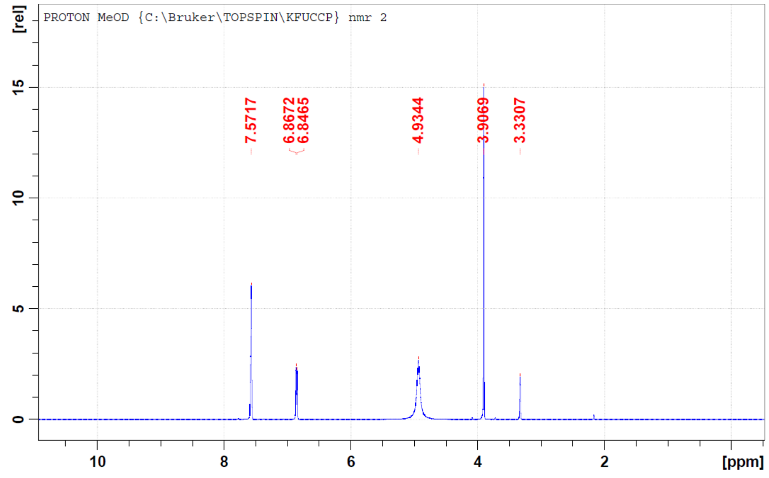

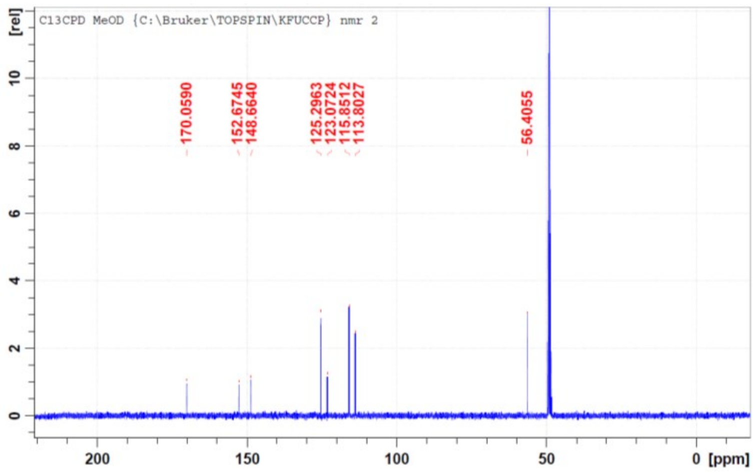

<u>^{1}H-NMR full spectrum of VA (400 MHz, CD_3OD)</u>

<u>^{13}C-NMR spectrum of VA (100 MHz, CD_3OD)</u>

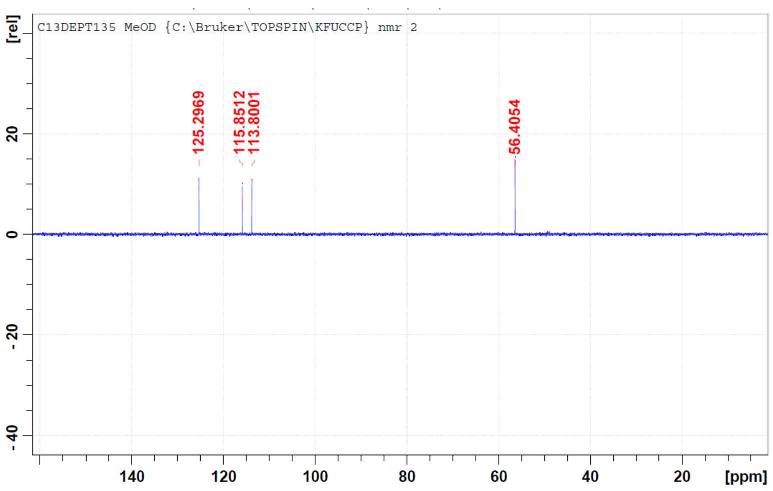

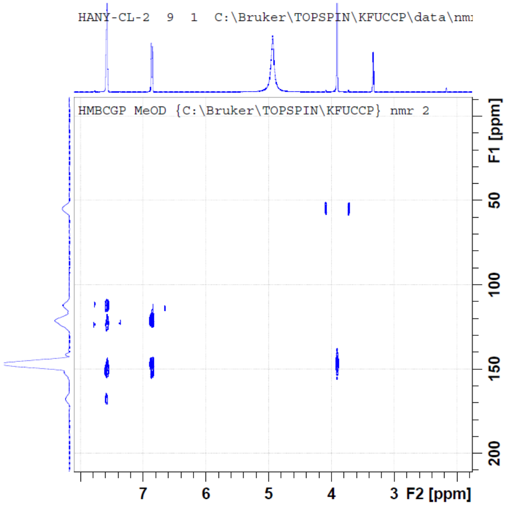

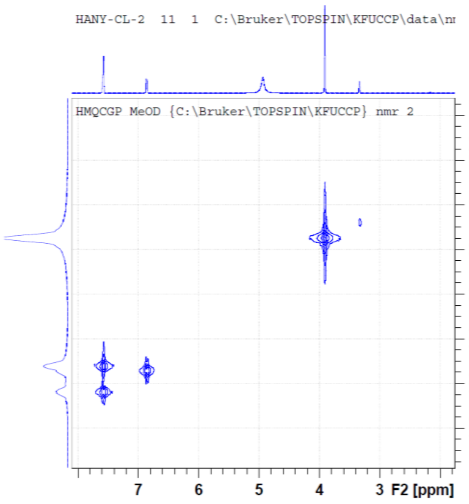

<u>DEPT spectrum of VA (100 MHz, CD_3OD)</u>

<u>HMBC spectrum of VA</u>

<u>HMQC spectrum of VA</u>

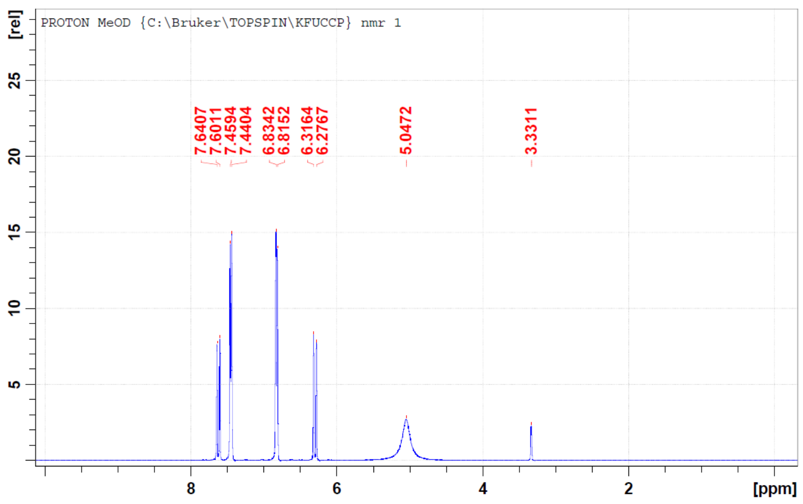

^{1}H-NMR full spectrum of PCA (400 MHz, CD_3OD)

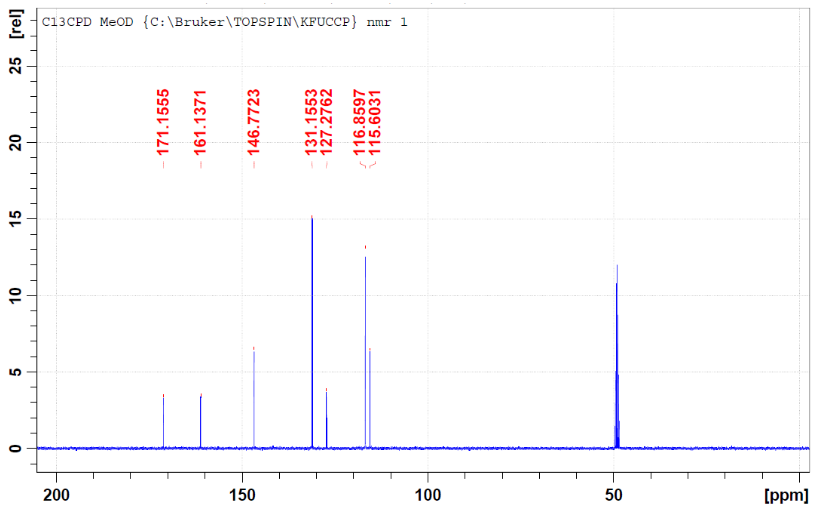

^{13}C-NMR spectrum of PCA (100 MHz, CD_3OD)

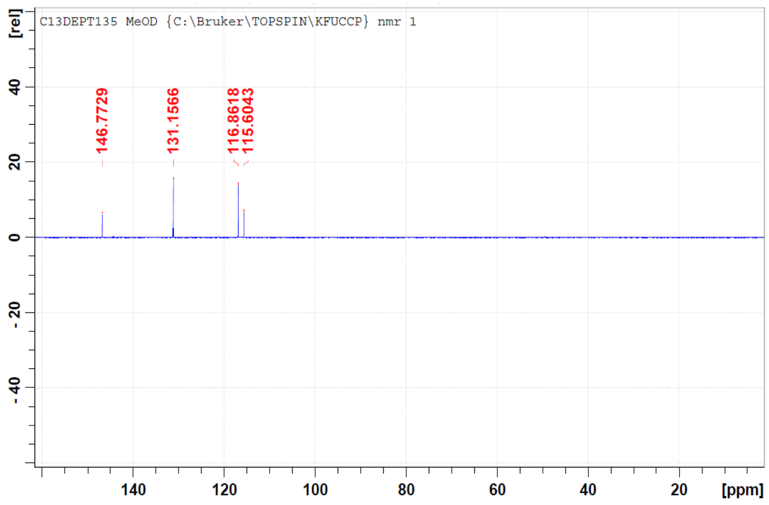

DEPT spectrum of PCA (100 MHz, CD_3OD)

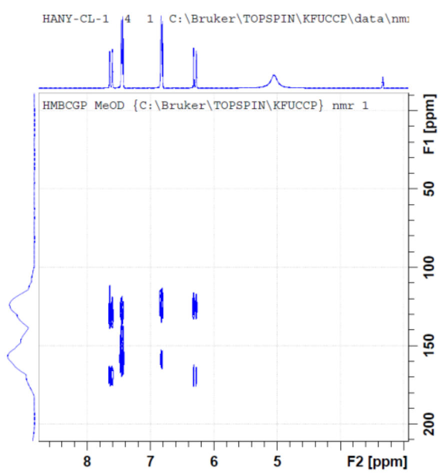

HMBC spectrum of PCA

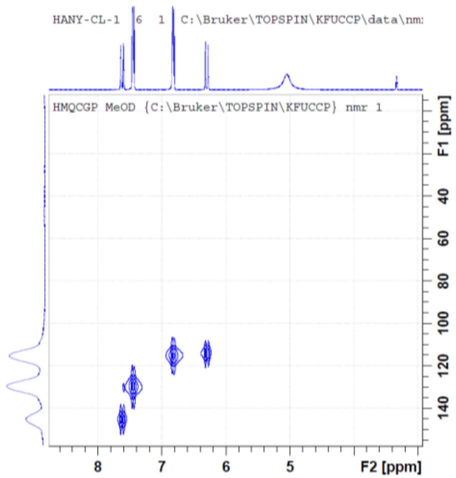

HMQC spectrum of PCA

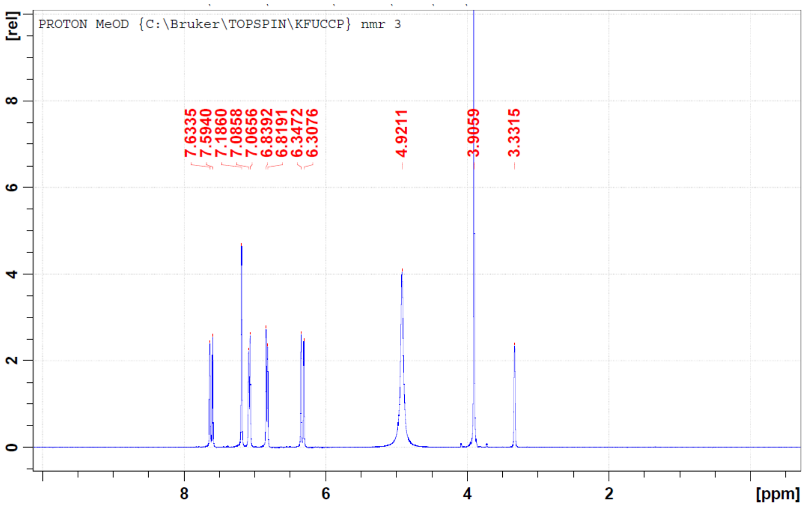

^{1}H-NMR full spectrum of FA (400 MHz, CD_3OD)

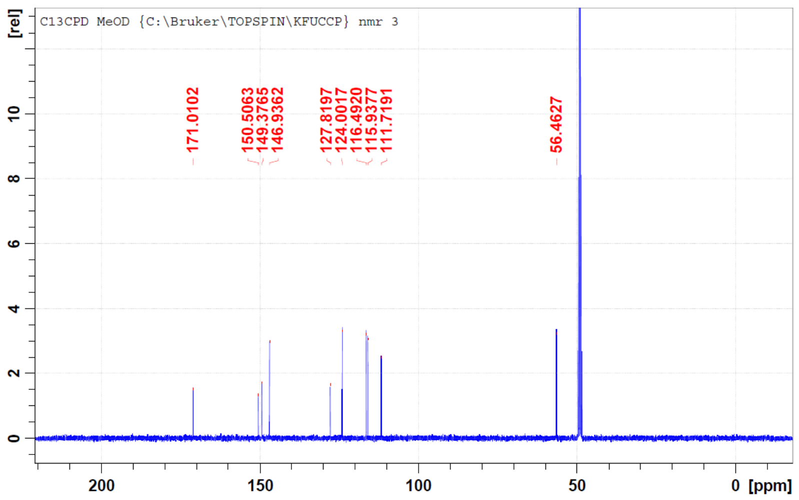

^{13}C-NMR spectrum of FA (100 MHz, CD_3OD)

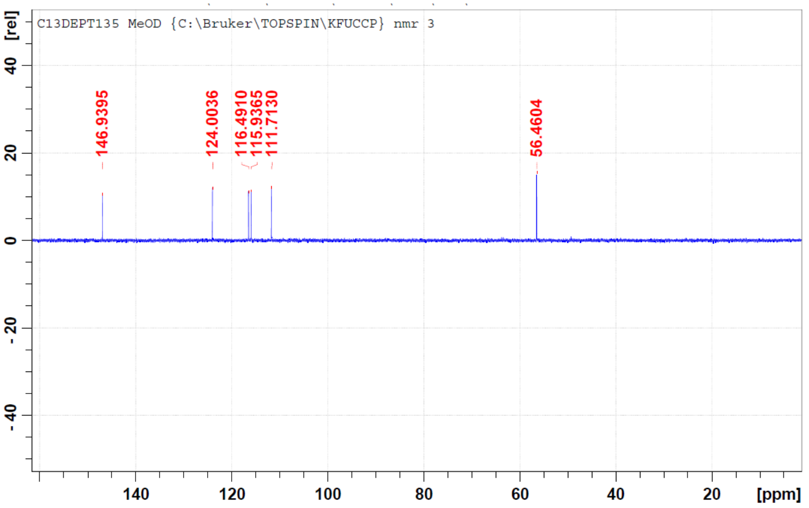

DEPT spectrum of FA (100 MHz, CD_3OD)

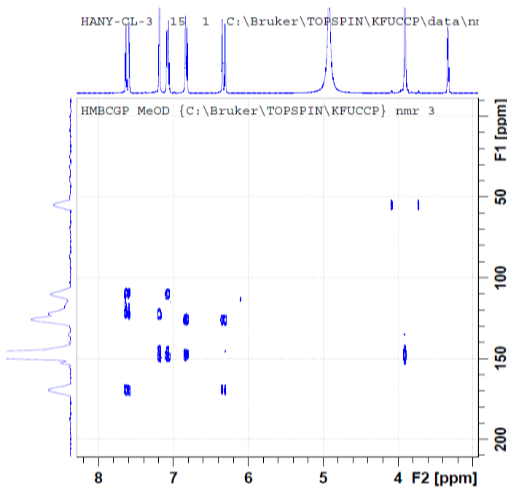

HMBC spectrum of FA

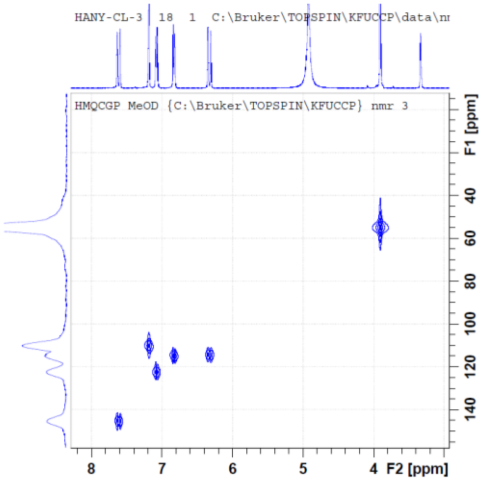

HMQC spectrum of FA

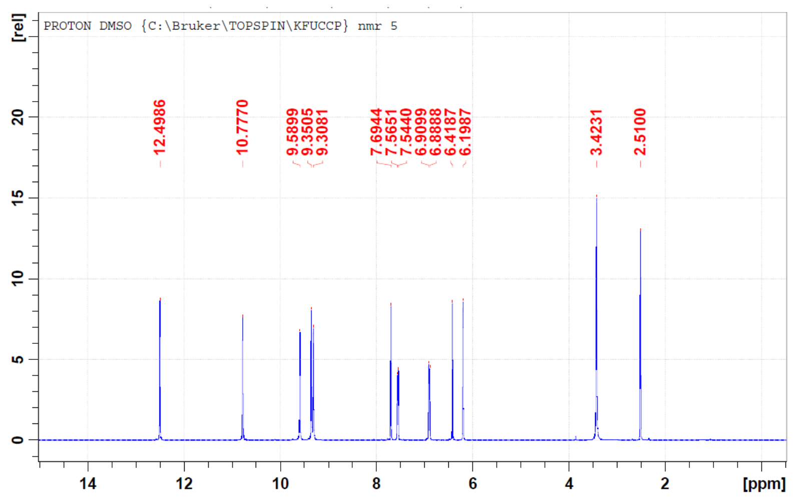

^{1}H-NMR full spectrum of QRN (400 MHz, DMSO-*d6*)

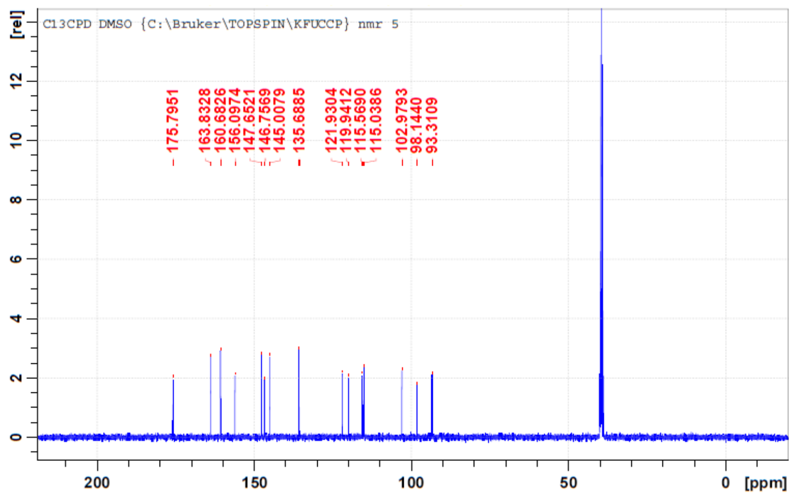

^{13}C-NMR spectrum of QRN (100 MHz, DMSO-*d6*)

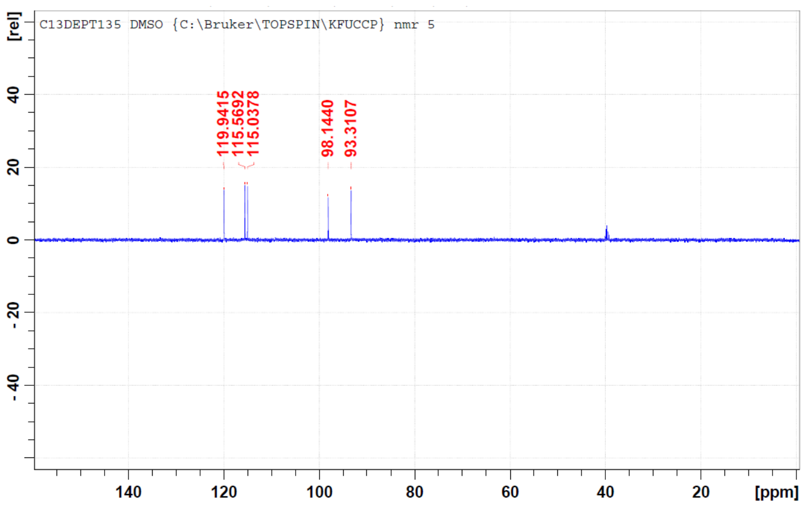

DEPT spectrum of QRN (100 MHz, DMSO-*d6*)

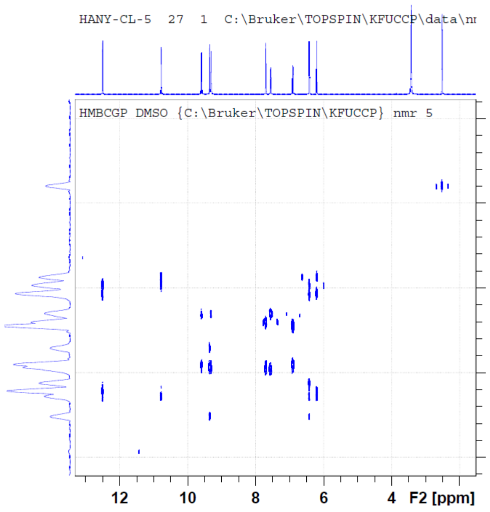

HMBC spectrum of QRN

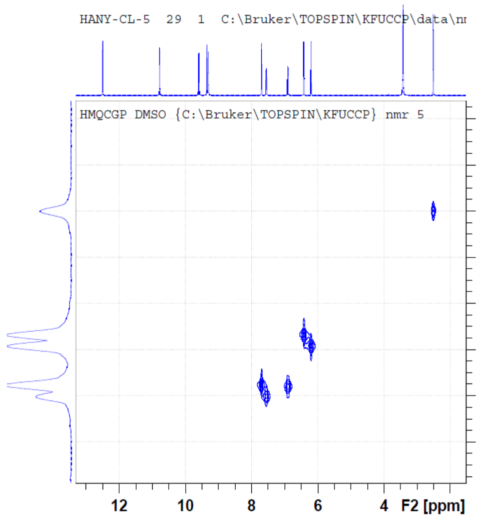

HMQC spectrum of QRN
